# Dual Task Effects on Speed and Accuracy During Cognitive and Upper Limb Motor Tasks in Adults With Stroke Hemiparesis

**DOI:** 10.3389/fnhum.2021.671541

**Published:** 2021-06-17

**Authors:** Hogene Kim, Hyun-Ki Kim, Nayoung Kim, Chang S. Nam

**Affiliations:** ^1^Department of Clinical Rehabilitation Research, National Rehabilitation Center, Seoul, South Korea; ^2^Department of Industrial Engineering, North Carolina State University, Raleigh, NC, United States

**Keywords:** stroke, cognitive motor interference, dual task, upper limb, movement, speed-accuracy trade-off

## Abstract

**Background:**

Adults with stroke need to perform cognitive–motor dual tasks during their day-to-day activities. However, they face several challenges owing to their impaired motor and cognitive functions.

**Objective:**

This case-controlled pilot study investigates the speed and accuracy tradeoffs in adults with stroke while performing cognitive–upper limb motor dual tasks.

**Methods:**

Ten adults with stroke and seven similar-aged controls participated in this study. The participants used a robotic arm for the single motor task and participated in either the serial sevens (S7) or the controlled oral word association test (COWAT) for single-cognitive task. For the dual task, the participants performed the motor and cognitive components simultaneously. Their speed and accuracy were measured for the motor and cognitive tasks, respectively.

**Results:**

Two-sample t-statistics indicated that the participants with stroke exhibited a lower motor accuracy in the cross task than in the circle task. The cognitive speed and motor accuracy registered by the subjects with stroke in the dual task significantly decreased. There was a negative linear correlation between motor speed and accuracy in the subjects with stroke when the COWAT task was performed in conjunction with the cross task (ρ = −0.6922, *p* = 0.0388).

**Conclusions:**

This study proves the existence of cognitive–upper limb motor interference in adults with stroke while performing dual tasks, based on the observation that their performance during one or both dual tasks deteriorated compared to that during the single task. Both speed and accuracy were complementary parameters that may indicate clinical effectiveness in motor and cognitive outcomes in individuals with stroke.

## Introduction

The successful recovery of upper limb (UL) sensorimotor functions allows survivors of hemiparetic stroke to perform daily activities without significant discomfort (Harris and Eng, 2010; Eraifej et al., 2017; Valdes et al., 2020). UL motor activities are more cognitively initiated and driven than activities such as walking, i.e., autonomous movements (Houwink et al., 2013). Modern UL therapies have adopted robotic technologies (Kwakkel et al., 2008) that occasionally demand the application of the visuo-cognitive and UL motor resources of the individuals with stroke. It has been observed that cognitive–motor dual tasks often resulted in cognitive–motor interference instead of motor or cognitive facilitation (Plummer and Eskes, 2015; Shin et al., 2017). Cognitive tasks considerably affect the motor function of the UL during robot-guided movements, thereby proving the presence of cognitive–motor interference (Shin et al., 2017).

Fitts’ law (Fitts, 1954) explains various human movement characteristics in terms of speed–accuracy tradeoffs (SATs). It claims that the speed of a movement is inversely related to its accuracy. SATs have been consistently used as a parameter in clinical studies that analyze human motor task performance by focusing on either the emphasis of speed (fast and inaccurate) or accuracy (slow and accurate)(Glenn and Parsons, 1991; Vallesi et al., 2012). In the field of neuro-rehabilitation, recent studies have reported the SATs as a possible parameter for the clinical assessment that estimated decreased capabilities in UL motor skill learning in patients with neurological disorders such as stroke (Fan et al., 2017; Kantak et al., 2018; Doost et al., 2019) and traumatic brain injuries (Korman et al., 2018). These studies evaluated patient’s neurophysiological changes that were successfully described in terms of SATs while conducting the paretic arm movement tasks, which were compared with outcomes in healthy controls.

Many neuropathological UL movements display decreased functional characteristics on SATs in individuals with upper motor neuron disorders, such as Parkinson disease (Fernandez et al., 2018), multiple sclerosis (Ternes et al., 2014), Huntington disease (Despard et al., 2015), and cerebral palsy (Davies et al., 2014; Fernani et al., 2017). For example, one study demonstrated that UL paretic movements were faster in contrast to their associated low accuracy during the movement task (Fernandez et al., 2018). A stroke, however, is known to be accompanied by mild to severe cognitive impairments, unlike the neuromuscular diseases mentioned above (Esmael et al., 2021). A hemiparetic stroke tends to cause both motor and cognitive impairments, thereby making it difficult to perform dual tasks that require cognitive resources. In fact, many day-to-day UL activities involve cognitive–motor dual tasks; for instance, typewriting involves simultaneous reading and typing, and it is therefore a visuomotor cognitive–motor task (Yamaguchi et al., 2013). Consequently in rehabilitation clinics, it would be of practical and clinical importance to utilize the dual task paradigm during extensive UL motor rehabilitation. However, to the best of our knowledge, there are limited studies that have investigated the execution of a cognitive–UL motor dual task by people with stroke hemiparesis, and has not previously been explored about the effectiveness of assessing SATs during a cognitive–UL motor dual task. Furthermore, most studies on modern robotic and visuo-cognitive technologies in motor rehabilitation have been increasingly applied to gait-driven dual task paradigm on lower limb motor rehabilitation (Subramanian et al., 2010; Ricklin et al., 2018), not on UL visuomotor cognitive dual task.

The purpose of this study is to investigate the speed and accuracy of a person with stroke while performing a cognitive–UL motor dual task. The first hypothesis that was tested stated that adults with stroke display significantly lower speed and accuracy, and mutual interference during a UL motor and cognitive dual task compared to those displayed by them during a single task. The second hypothesis claimed that, while performing a dual task, the cognitive and motor outcomes of the adults with stroke were less accurate in comparison to those of healthy controls. We analyzed the effects of the motor and cognitive components of the single and dual tasks on the speed and accuracy of the subjects with stroke and healthy controls of similar ages.

## Materials and Methods

### Subjects

Ten adults with chronic stroke (54.7 ± 12.3 years; M: 10) and seven age- and gender-matched controls were recruited; they were asked to perform a series of cognitive–motor tasks. A power analysis using G^∗^Power 3.1.9.4 (Faul et al., 2007) for an independent-sample *t*-test was conducted assuming one-tailed testing with a large effect size of *d* = 1.3, 80% power and alpha error probability of a= 0.05 (McGough and Faraone, 2009; Taub et al., 2011). This analysis suggested a total sample size of at least 10 subjects with stroke and eight healthy controls. This study was approved by the institutional review board of the National Rehabilitation Center, Seoul, South Korea, and registered clinical human subject registry (cris.nih.go.kr registration: KCT0004873). The written informed consent forms of all the participants were obtained before collecting data. The study followed all STROBE guidelines and reported the necessary information appropriately (see Supplementary Video 1).

The eligibility criteria for the participants were given based on the previous studies, and shown as follows: (1) first-time and chronic poststroke hemiparesis (>3 months); (2) > 18 years of age; (3) manual muscle testing (MMT) at shoulder and elbow joints is above the poor grade (Zero/Trace/Poor/Fair/Good/Normal scale) (Cuthbert and Goodheart, 2007); (4) Modified Ashworth scale (MAS) at upper extremities less or equal than 1+ (0/1/1+/2/3/4 scale)(Pandyan et al., 1999); and (5) mini-mental states examination (MMSE) over than 23 (24–30: No cognitive impairment, 18–23: Mild cognitive impairment, 0–17: Severe cognitive impairment)(Zwecker et al., 2002). Individuals with the following conditions were excluded from the study: (1) stroke with multiple or bilateral lesions; (2) recurrent stroke; (3) complications of orthopedic disorders; (4) communication disorders due to aphasia; and (5) mental illnesses.

### Single Tasks

**FIGURE 1:**
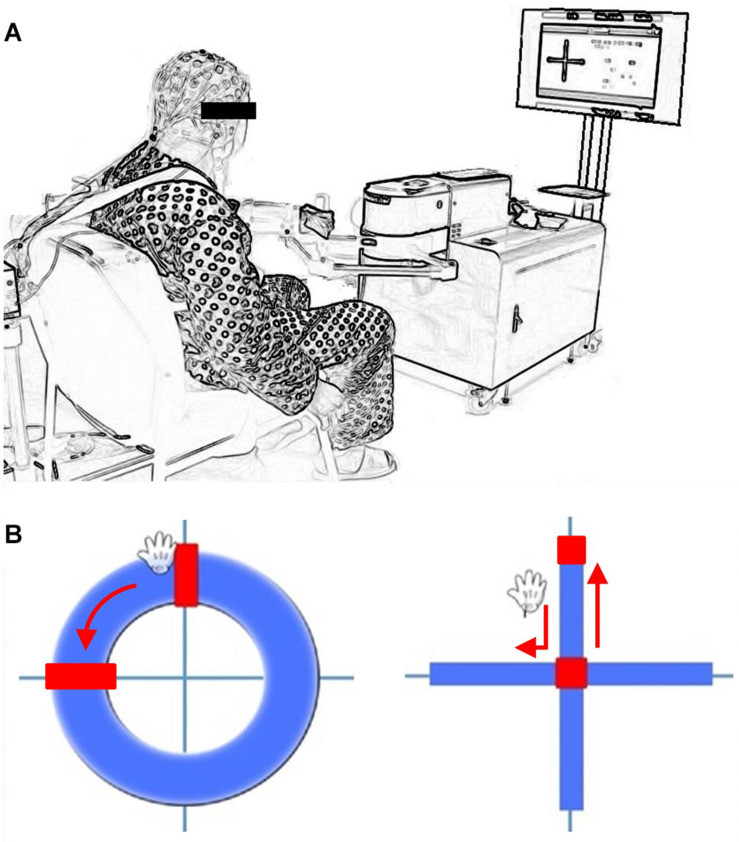
**(A)** Cognitive-Upper Limb Motor dual task test setup (3DBT-63, Man&Tel Inc., Gumi, South Korea) **(B)** Visual feedback with movement cursor and direction indicator (Circle and Cross movement task).

The upper limb movements were performed using customized commercial upper limb robotic rehabilitation equipment for the single-motor task, as shown in Figure 1A (Camillo 3DBT-61, Man&Tel Inc, South Korea). The participants were seated in a comfortable chair and fastened to it with a trunk seatbelt to minimize any additional compensatory movement and prevent an accidental fall. The subject’s paretic arm was fastened to a handle with an upper arm support by using the Velcro provided in the equipment. The subjects were required to move a cursor by using the robotic arm to follow a moving red target in the feedback monitor within the designated areas (circle or cross-shapes) and testing time (1 min) for the single task. In the task involving the circular shape, the subject was required to move the cursor along an annulus by using the affected hand. The cross-shaped task required the participants to perform center-in and center-out movements in four clockwise and counter-clockwise directions, as shown in Figure 1B. In many modern robotic UL motor equipment, the cross-shaped reaching task was adopted for the UL movement tasks as well as linear- and circular-shaped movements (Brewer et al., 2007). The single-cognitive task consists of a serial sevens subtraction test (S7) that involves the serial subtraction of seven from a randomly chosen three-digit number (for instance, subtracting 7 from 203) or a controlled oral word association test (COWAT) task that requires the subject to orally state related words as much as possible within 1 min (for instance, saying hospital-related words or words beginning with “B”). These S7 and COWAT tests have been widely used to test the diagnostic values about cognitive abilities of subjects with cognitive impairments in clinics (Milstein et al., 1972; Malek-Ahmadi et al., 2011; Cullen et al., 2019).

### Dual Task Effect

The subjects were required to perform UL motor tasks identical to those in the single tasks, in addition to performing the serial sevens or COWAT test simultaneously for one minute in the dual task paradigm. The dual task effect (DTE) is used to quantify the effects of the dual task performance on various parameters compared to the single task performance, as demonstrated by Plummer and Eskes [6]. The DTE is calculated as shown below:

$$\text{D}\,u\,a\,l\,T\,a\,s\,k\,E\,f\,f\,e\,c\,t\,\left(\text{D}\,T\,E\right)=\frac{\mathrm{Dual}\,\mathrm{task}\,\mathrm{performance}-\mathrm{Single}\,\mathrm{task}\,\mathrm{performance}}{\mathrm{Single}\,\mathrm{task}\,\mathrm{performance}}\times 100$$

### Speed and Accuracy

The verbal answers provided during the cognitive tasks were recorded from the beginning of each task while noting down the correct answers provided by the subjects. This was followed by the calculation of the cognitive speed, which is defined as the ratio of the total number of answers to the task time, and cognitive accuracy, which is defined as the ratio of the number of correct answers to the total answers provided in a task. Similarly, the movement trajectories were recorded during the motor task and used to calculate the motor accuracy and motor speed. The motor accuracy is equal to the percentage of movement trajectories within the annulus, and the motor speed is defined as the ratio of the total distance to the task time.

### Statistical Analysis

Descriptive statistics were applied to the motor and cognitive variables to depict the motor accuracy and the number of accurate answers. The hypotheses were tested by comparing the single and dual task performances through a paired two-sided *t*-test and an independent sample student *t*-test on the subjects with stroke and healthy controls. The SPSS Analytic Server Version 21.0.0.1 (IBM Corporation, Chicago, Illinois, United States) was used to perform the statistical analysis wherein the significance level was set at 0.05.

## Results

### Subjects

The 10 subjects with chronic stroke (average onset time: 50.9 months; seven subjects with left hemiparesis) participated in an initial screening (Fugl–Meyer assessment: upper extremity = 40.1 ± 16.5; mini-mental state examination = 28.9 ± 1.4) followed by the single and cognitive–UL motor dual tasks. Seven age- and gender-matched healthy control subjects also participated in the study. The subject’s demographics and clinical information is shown in Table 1.

**TABLE 1:** Subject Demographics.

	**ID**	**Age (yrs)**	**Ht (cm)**	**Wt (Kg)**	**Onset (mths)**	**Side (L/R)**	**MMSE**	**FMA-UEx**	**MAS**		**MMT**	
	
									**Elbow**	**Wrist**	**Shoulder**	**Elbow**
Subjects with stroke hemiparesis	1	61	160	62	10	L	27	49	0	0	F	F
	2	39	190	120	6	L	29	21	0	0	F	F
	3	61	174	74	87	R	30	55	0	0	F	F
	4	53	162	79	36	L	30	57	0	0	F	F
	5	77	167	62	201	L	29	55	0	0	F	F
	6	53	174	80	96	R	30	52	1	1+	F	F
	7	42	170	75	5	L	26	10	1	1	F	F
	8	68	162	63	10	L	30	30	1+	1	F	F
	9	52	177	78	39	L	28	29	1+	1+	F	F
	10	41	178	78	19	R	30	43	1	1	F	F
	mean	54.7	171.4	77.1	50.9	7 L	28.9	40.1	−		−	
	(sd)	(12.3)	(16.7)	(16.4)	(62.1)		(1.4)	(16.5)				
H.C.	mean	58.4	162.2	67.7	–	–	–	–	–		–	
(*n* = 8)	(sd)	(10.6)	(13.1)	(17.5)								

*H.C, healthy controls; MMSE, Mini-Mental State Examination. FMA-UEx, Fugl-Meyer Assessment of the Upper Extremity. MAS, Modified Ashworth Scale. MMT, Manual Muscle Test; F, Fair.*

### Single vs. Dual Tasks

As shown in Table 2, comparisons between the results of the single and dual tasks demonstrated that the motor accuracy of the subjects with stroke during the single motor task (circle only) was significantly lower than that observed during the dual task (circle + S7). The motor accuracies of the single and dual tasks were significantly decreased from 84.9% ± 11.2% to 79.0% ± 16.3%, respectively (*p* = 0.017). The motor accuracy of the subjects with stroke during the dual tasks (COWAT + cross) was also significantly reduced, with accuracies of 77.2% ± 13.2% and 74.6% ± 12.4% for the single and dual tasks, respectively (*p* = 0.034). The cognitive speed of the subjects with stroke during the dual task (S7 + cross) was significantly less than that observed during the single task. The cognitive speeds of the subjects with stroke during the single and dual tasks were equal to 11.3 ± 5.4 and 9.3 ± 4.7, respectively (*p* = 0.008).

**TABLE 2:** Cognitive and motor speed and accuracy in single and dual tasks between participants with stroke and healthy controls.

				**Healthy controls**	**Stroke subjects**	**Healthy controls**	**Stroke subjects**

				**Motor Task (O shape)**		**Motor Task (+ shape)**	
Motor Task	Speed (cm/sec)	Single Task		23.6 ± 12.3	15.9 ± 4.7	11.3 ± 4.7	11.0 ± 3.5
		Dual Task	S7	19.1 ± 9.8	14.6 ± 3.5	9.9 ± 4.0*	10.0 ± 3.2
			COWAT	17.5 ± 10.0*	15.1 ± 5.2	9.4 ± 3.6	11.1 ± 4.3
	Accuracy (%)	Single Task		93.1 ± 5.14	84.9 ± 11.2	88.6 ± 8.2	77.2 ± 13.2
		Dual Task	S7	92.9 ± 8.9	79.0 ± 16.3*†	87.0 ± 9.8	74.9 ± 13.5
			COWAT	91.8 ± 8.5	86.1 ± 10.7	90.3 ± 7.7*	74.6 ± 12.4*†

				**Cognitive Task (Serial 7)**		**Cognitive Task (COWAT)**	

Cognitive Task		Single Task		12.7 ± 6.3	11.3 ± 5.4	11.6 ± 3.6	11.0 ± 2.6
	Speed (answers/min)	Dual Task	O	13.3 ± 4.9	10.6 ± 5.8	10.2 ± 4.9	11.0 ± 2.2
			+	11.0 ± 5.6	9.3 ± 4.7*	11.1 ± 5.4	10.4 ± 2.6
	Accuracy (%)	Single Task		79.6 ± 14.7	86.8 ± 9.5	95.3 ± 3.1	95.5 ± 4.7
		Dual Task	O	80.2 ± 20.2	88.4 ± 12.1	93.9 ± 7.9	97.1 ± 3.7
			+	75.7 ± 20.4	84.3 ± 10.8	90.8 ± 9.1	96.4 ± 3.6

S7 denotes “Serial 7” cognitive task. COWAT denotes a controlled oral word association test; O denotes motor task with circle shape movement track. + denotes a motor task with cross-shaped movement track. *p-value by paired *t*-test between single and dual task results. **^†^***p*-value by student’s *t*-test between stroke and control. Bold digits indicate values with statistical significance (*p* < 0.05).

### Stroke vs. Control

The paired-t statistics in Table 2 indicated that the motor accuracy of the stroke subjects was significantly lowered during the cross component of the single task than it was during the circle component (*p* = 0.006). There was no significant change in the number of correct answers and the motor accuracy of the control subjects during the single and dual tasks.

However, there was a significant difference in the movement accuracies between the subjects with stroke and the healthy controls during the dual tasks (Table 2). The subjects with stroke and the healthy controls registered movement accuracies of 79.0% ± 16.3% and 92.9% ± 7.9% during the Circle + S7 dual task, respectively (*p* = 0.035). Similarly, the subjects with stroke and the healthy controls reported movement accuracies of 74.6% ± 12.4% and 90.3% ± 7.7% during the Cross + COWAT dual task, respectively (*p* = 0.010).

There was a trend that the motor accuracy of the stroke group was lower than that of the control group, especially during single-motor conditions. The movement accuracies of the stroke and control subjects during the circle test were 84.9% ± 11.2% and 93.1% ± 5.1%, respectively (*p* = 0.094). Similarly, the movement accuracies of the stroke and control subjects during the cross test were 77.2% ± 13.2% and 88.6% ± 8.2%, respectively (*p* = 0.062). No significant difference was found between the controls and the subjects with stroke during the single-cognitive task.

### Dual Task Effects

**FIGURE 2:**
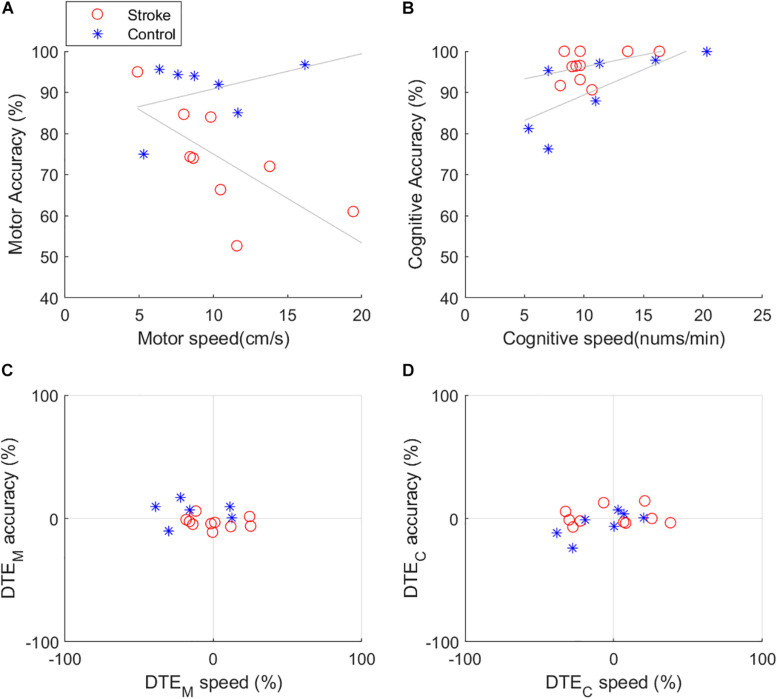
Dual task effects on speed and accuracy during cognitive (COWAT) and upper limb motor (Cross) dual task **(A)** Speed and accuracy relationship in motor task **(B)** Speed and accuracy relationship in cognitive task **(C)** DTE motor speed and accuracy **(D)** DTE cognitive speed and accuracy.

Dual task effects were shown between the results of speed and accuracy during cognitive and motor dual task described in Table 3 and Figure 2. DTE in motor accuracy and cognitive speed during dual task of Circle and Serial 7 was significantly lower motor accuracy (*p* = 0.034) and cognitive speed (*p* = 0.032) in individuals with stroke. Cognitive speed and accuracy were significantly deteriorated in healthy controls during cognitive (Serial 7) motor(cross) dual task. (cognitive speed *p* = 0.039; cognitive accuracy *p* = 0.046) compared to corresponding single task outcomes. There was a significant negative linear correlation between motor speed and motor accuracy in subjects with stroke (ρ = −0.6922, *p* = 0.0388) (Figure 2A).

**TABLE 3:** Dual Task Effects in Motor and Cognitive Tasks.

	**DTE**			**O**			**+**		
**Motor Task**				**Healthy**	**Stroke**	***p*-value†**	**Healthy**	**Stroke**	***p*-value†**
	Speed (cm/sec)	Dual task	S7	−16.1 ± 17.4	−4.9 ± 17.1	0.213	−10.1 ± 14.3	−6.9 ± 17.0	0.678
			COWAT	−18.8 ± 19.8	−8.4 ± 14.3	0.261	−13.0 ± 19.5	−0.1 ± 15.9	0.173
			*p*-value	0.786	0.708	−	0.527	0.153	–
	Accuracy (%)	Dual task	S7	−0.3 ± 3.4	−**7.8 ± 9.0**†	**0.034**	0.1 ± 7.1	−2.5 ± 10.3	0.540
			COWAT	−1.6 ± 4.6	1.8 ± 8.7*	0.312	3.5 ± 6.0	−2.1 ± 7.5	0.106
			*p*-value	0.120	**0.028**	–	0.382	0.926	–

Cognitive Task				**Serial 7**			**COWAT**		
				**Healthy**	**Stroke**		**Healthy**	**Stroke**	

	Speed (answers/min)	Dual task	O	11.0 ± 18.4	−**9.3 ± 14.2**†	**0.032**	−13.4 ± 30.1	3.0 ± 22.6	0.246
			+	−12.3 ± 21.0*	−16.8 ± 23.8	0.684	−7.8 ± 20.9	−2.0 ± 25.5	0.611
			*p*-value	**0.039**	0.280	−	0.597	0.286	–
	Accuracy (%)	Dual task	O	12.9 ± 36.3	−6.4 ± 18.0	0.228	7.5 ± 27.8	−4.3 ± 23.3	0.372
			+	−17.6 ± 19.7*	−19.7 ± 18.7	0.832	−1.9 ± 27.8	−0.1 ± 25.6	0.896
			*p*-value	**0.046**	0.109	–	0.384	0.412	–

*S7 denotes “Serial 7” cognitive task. COWAT denotes; O denotes a motor task with circle shape movement track. +, denotes a motor task with cross-shaped movement track; Bold, significant *p*-value (*p* < 0.05). ^∗^*p*-value by paired *t*-test between single and dual task results. ^†^*p*-value by student’s *t*-test between stroke and control.*

## Discussion

In this pilot study, we proved that the individuals with stroke demonstrated speed-accuracy tradeoffs during a cognitive and UL motor dual task, in conjunction with a greater emphasis on deteriorated cognitive performance. The people with stroke exhibited a lower motor accuracy compared to that of the healthy controls. However, the cognitive accuracies of the subjects with stroke and healthy controls were similar during the dual tasks. The people with stroke displayed a tendency to sacrifice motor accuracy to sustain motor speed and cognitive performance. This is contrary to the theory of sacrificing speed for accuracy in motor tasks, as stated by Fitts’ Law. However, the healthy controls sacrificed motor speed and produced similar outcomes. It was demonstrated that speed and accuracy were the appropriate parameters for describing the outcome differences in cognitive and UL motor dual tasks between people with stroke and healthy controls.

To perform either a fine motor or challenging cognitive task, exerted cognitive efforts to increase or sustain the accuracy may exist. The people with stroke displayed a significantly lower UL motor accuracy compared to the healthy controls during dual tasks (Table 1). This is an acceptable outcome because the hemiparetic movements of subjects with stroke are assumed to be less accurate than those of the healthy participants. The validity of these results was verified by previous studies that have demonstrated the deterioration of the motor capabilities of subjects with stroke while performing cognitive–motor dual tasks, particularly during lower extremity activities such as walking (Bowen et al., 2001; Plummer-D’Amato et al., 2008) or balancing (Bensoussan et al., 2007). These studies also stated that subjects with stroke, unlike the healthy controls, prioritized cognitive tasks, such as maintaining the walking speed, center of pressure, and double support time, when the motor outcomes are significantly affected. Because the primary focus of this study was to analyze the deteriorated motor outcomes due to divided cognitive attention, we have not compared the effects of the single and dual tasks on the motor speed and accuracy of the UL. However, the healthy controls registered a better motor accuracy during the dual task than they did during the single task (Table 1). These results prove that the singular and dual natures of the motor and cognitive tasks affect the functional outcomes of the speed and accuracy paradigm differently. In the rehabilitation clinics, for examples, the motor accuracy during dual task may directly indicate the progress in UL motor rehabilitation, which potentially predict future performances of adults with stroke on several UL-involved and cognitive-driven daily activities while living in the community.

The DTE demonstrated that each group applied a different strategy while performing the dual circle (autonomous movement) and cross (cognitively-driven movement) motor tasks. There was no significant difference between the DTE values of the people with stroke and those of the healthy controls during the cross component of the dual task. However, a significant difference was observed in the DTE values of the people with stroke and those of the healthy controls while performing the circle component of the dual task. The people with stroke displayed more interference than the healthy controls in terms of motor accuracy, while the latter facilitated cognitive accuracy. Dual tasks related to walking have been an area of focus in stroke rehabilitation studies. These studies have obtained dual task outcomes that are similar to those obtained in the current study; for instance, they observed postural unsteadiness while demanding attention during walking, which is an autonomous movement (Brown et al., 1999; Yang et al., 2007). Similarly, the cognitive demands associated with the (autonomous) UL movements during the circle component may be lower than those of the UL (cognitive-driven) movements during the cross component of the cognitive–motor dual tasks. The different DTE values indicate that healthy controls are more likely to prioritize motor accuracy, which demands more cognition, compared to people with stroke. People with stroke are less likely to demand cognitive resources to improve their motor accuracy during a dual task because of their cognitive impairments. Therefore, they require more cognitive resources to increase their motor accuracy This study has successfully explored about the effectiveness of assessing SATs during a cognitive–UL motor dual task in adults with stroke. The SATs assessment may provide useful clinical information on UL motor rehabilitation, particularly when applying modern robotic and visuo-cognitive technologies.

Speed and accuracy are complementary parameters to effectively examine the task performance outcomes in people with stroke during cognitive and task-specific UL motor dual tasks. Researchers have encountered several unresolved issues indicating that people with stroke have a higher risk of mild cognitive impairments and dementia (Knopman et al., 2009). However, mild cognitive impairments in people with stroke have been shown to decrease UL dual-task performance (Toosizadeh et al., 2016). The cause of the decreased performance in dual tasks has been investigated in terms of executive and neurophysiological dysfunctions in people with mild cognitive impairments (Johns et al., 2012; Kirova et al., 2015). In general, tasks that require executive attentional resources have been shown to adversely affect the task performance outcomes (Brown et al., 2015). Therefore, unlike gait training (i.e., autonomous movements), an effective training method for UL movements in people with stroke may concurrently affect other UL movements. A previous study on task-specific UL training methods successfully demonstrated that one trained UL movement task (i.e., feeding) potentially had lasting therapeutic effects on two untrained tasks, (i.e., sorting and dressing) (Schaefer et al., 2013). Therefore, a training paradigm that provides cognitive and UL motor dual-tasks would be appropriate for stroke UL movement rehabilitation; measurements of speed and accuracy provide useful information concerning a patient’s rehabilitation.

In the future, a study should be conducted to investigate the clinical effectiveness in comprehensive interventions of cognitive and UL motor dual tasks for people with stroke who exhibit limited UL functions. The current study was preliminary which had limited samples of a gender-biased small number of participants. Future studies should involve an increased number of subjects and an investigation into task-specific motor activities such as the level of difficulty of the motor and cognitive tasks.

In summary, people with stroke were observed to have a UL motor accuracy during cognitive and UL motor dual tasks instead of a slow motor speed. Speed and accuracy were used as complementary parameters that may be capable of effectively indicating clinical progress in motor and cognitive rehabilitation outcomes.

## Conclusion

In this study, we demonstrated that a cognitive–UL motor interference occurs in people with stroke hemiparesis while performing dual tasks; this was based on the observations of their performances with respect to speed and accuracy during single and dual tasks. Dual task effects indicated a deterioration in performance in the dual tasks compared to that of the single task. Speed and accuracy are the complementary parameters that may indicate clinical effectiveness in motor and cognitive outcomes in people with stroke.

## Data Availability Statement

The original data presented in the study are included in the Supplementary Material.

## Ethics Statement

The studies involving human participants were reviewed and approved by Institutional Review board at National Rehabilitation Center, Seoul, South Korea. The patients/participants provided their written informed consent to participate in this study. Written informed consent was obtained from the individual(s) for the publication of any potentially identifiable images or data included in this article.

## Author Contributions

HK, H-KK, NK, and CSN had equal contributions to the conception, design and methodology of the work, data acquisition, supervision, and project administration. H-KK and HK contributed to analyze the data, validation, formal analysis, and visualization. HK contributed to original draft preparation, review, editing, and funding acquisition. All authors have read and agreed to the published version of the manuscript.

## Conflict of Interest

The authors declare that the research was conducted in the absence of any commercial or financial relationships that could be construed as a potential conflict of interest.
