# Supplementary material for: Dual Task Effects on Speed and Accuracy During Cognitive and Upper Limb Motor Tasks in Adults With Stroke Hemiparesis
Source: Front Hum Neurosci. 2021 Jun 17;15:671541. doi: 10.3389/fnhum.2021.671541 (PMC8250862; doi:10.3389/fnhum.2021.671541)
Supplement: Supplementary file 2 [file Table_1.DOCX]

**STROBE Flow Diagram**

## Enrollment

## Allocation

## Analysis

Analysed (n=17)
♦ Excluded from analysis (n=0)

Allocated to clinical trial (n=17)

♦ Subject eligible for allocated trials (n=17)

♦ Did not receive allocated trial (n=0)

♦  Excluded because of exclusion criteria (n=0)

− Unable to move upper limb (n=0)

− Unable to follow instructions (n=0)

Total available subjects (n=17)
